# Association between Physical Frailty and Sleep Quality among Saudi Older Adults: A Community-Based, Cross-Sectional Study

**DOI:** 10.3390/ijerph182312741

**Published:** 2021-12-03

**Authors:** Bader A. Alqahtani

**Affiliations:** Department of Health and Rehabilitation Sciences, Prince Sattam Bin Abdulaziz University, Al-Kharj 11942, Saudi Arabia; dralqahtaniba@gmail.com; Tel.: +966-580422762; Fax: +966-0115886328

**Keywords:** frailty, sleep quality, Saudi, prefrailty, older adults

## Abstract

(1) Background: Prevalence of poor sleep quality and its association with frailty status among the aging population of Saudi Arabia has not been studied. Therefore, the main objective of the current study was to estimate the prevalence of poor sleep quality and investigate the association between poor sleep quality and frailty in Saudi older adults; (2) Methods: A total of 270 (mean age 69.9 ± 6.2) older adults from the Riyadh region were involved in the study. To measure sleep quality, the Arabic version of the Pittsburgh Sleep Quality Index (PSQI) was used. The Fried’s frailty index was utilized to assess frailty. Using multiple logistic regression models, the association between sleep quality and frailty status was evaluated using the Odds Ratio and confidence intervals (CI 95%); (3) Results: The pre-frailty and frailty status were prevalent among older adults who had poor sleep quality, 37% and 37.6% (*p* < 0.001), retrospectively. Poor sleep quality (PSQI > 5) was independently associated with both frailty (OR = 2.13) and prefrailty groups (OR = 1.67); (4) Conclusions: our study demonstrated a significant association between frailty and poor sleep quality. However, a longitudinal future study needs to be established to confirm this association and establish the causality relationship.

## 1. Introduction

Saudi Arabia’s elderly population will rise over the next few decades. According to UN projections, the Saudi elderly population will grow by 22.9% by 2050 compared to 5.6% in 2017 [1]. With an increasing number of elderly people in Saudi Arabia, the health-care system faces a heavier burden considering the high prevalence of chronic conditions that need close monitoring and treatment, including diabetes, arthritis, cardiovascular disease, and aging-related problems, such as frailty [2,3].

Aging has already been related to declines in several bodily functions, including musculoskeletal, cardiovascular, sensory, and cognitive functions [4]. Furthermore, frailty has been related to aging, which often leads to an increased risk of falling, sensitivity to poor health outcomes, functional limitations, and subsequent long-term care or nursing home admission [5,6]. Because of the present significant increase in the amount of senior people in Saudi Arabia, the subject of frailty and the related health consequences are more relevant than ever [7].

Frailty is described as a clinical geriatric condition that is characterized by an increased vulnerability to adverse outcomes [8]. Frailty has emerged as a major public health concern in the geriatric population [9]. According to a meta-analysis of research from various populations, frailty prevalence ranged from 4% to 51% in China and Cuba, respectively [10]. In addition, a recent research reported 21% prevalence of frailty among older adults in Saudi Arabia [11,12,13]. Furthermore, research suggests that frailty is a dynamic condition and may be controlled and prevented by treatments, such as health promotion, diet, physical activity, and social engagement [14]. As a result of the aging population’s rapid expansion, greater emphasis should be placed on identifying risk factors and avoiding frailty in elderly individuals with chronic illnesses.

Poor sleep quality and sleep efficiency become more prevalent among older adults [15,16]. Sleep problems have an adverse impact on the general health of older adults. Many research studies have found an association between poor sleep quality in the elderly and a higher 5-year mortality, reduced quality of life, higher risk of cardiovascular conditions, and poor cognitive function [17,18,19]. As a result, sleep disorders in adults have emerged as a new topic to investigate via the lens of preventative rehabilitation intervention.

Sleep disturbances are among the issues that have lately been investigated for their potential role in frailty. Previous studies have shown that frailty can disturb the sleep cycle and have hypothesized a bidirectional link between frailty and sleep disorders [20,21]. The association between frailty and sleep disturbances has been established in different populations in different countries. However, studies that have examined the sleep parameters and geriatrics syndrome, i.e., frailty among Saudi elderly, is lacking. Given the negative effect of sleep disturbances on health outcomes and the increase in geriatrics population in Saudi Arabia, studying the prevalence of sleep disturbances and its association with other geriatric conditions, such as frailty, is paramount. Therefore, the present study aimed to estimate the prevalence of poor sleep quality and examine the relationship between sleep quality and frailty among Saudi community-dwelling older individuals. We hypothesized that physical frailty was associated with poor sleep quality among Saudi older adults.

## 2. Materials and Methods

### 2.1. Study Design and Sample

**Figure 1:**
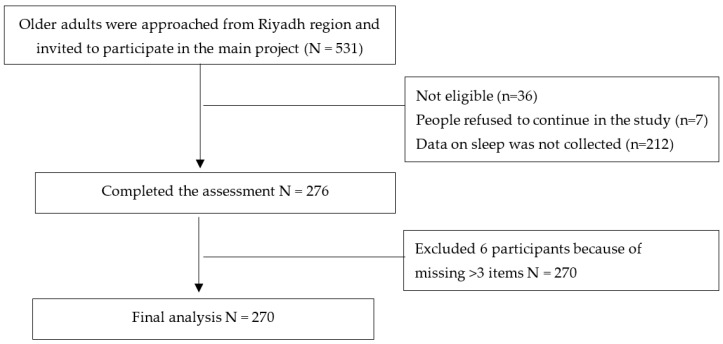
Flow chart of participants’ enrollment.

This is a cross-sectional study part of an ongoing project that includes older adults residing in the community who are aged 60 years and older and residing in the Riyadh region. This research was conducted from August 2019 to June 2020. This study was part of a previous project where measurements of sleep was collected on a subsample of the original study sample. An explanation of the sampling and recruitment methods of the original sample can be found elsewhere [11]. Detail about participants’ enrollment is shown in Figure 1.

All participants provided written informed consent before enrolling in the study. An ethical approval from the ethical committee at Prince Sattam bin Abdulaziz University was obtained. The current study adheres to the Helsinki Declaration criteria for medical research involving human participants.

### 2.2. Inclusion and Exclusion Criteria

Participants were considered if they were over the age of 60. Those participants with any acute illness or unstable health problem that would impair their capacity to complete the included outcome measures were excluded from the analysis.

### 2.3. Outcome Measures

#### 2.3.1. Frailty

The Fried’s frailty phenotype was utilized to assess frailty in the current study. Scoring was defined based on the presence of the below criterions:

1. Unintentional weight loss. This component was assessed by a self-report of unintentional weight loss of 10 pounds or more in the previous year.
2. Exhaustion. This component was measured by answering two questions from the Center for Epidemiological Studies Depression scale, “I felt that everything I did was an effort” and “I could not get going”.
3. Slowness. This component was defined by time taken to walk 15 feet (4.57 m), taking into account both gender and height.
4. Weakness. Grip strength was measured, stratified by gender and Body Mass Index (BMI).
5. Low physical activity. This component was defined as the rate of energy expenditure weekly, using questions from the Minnesota Leisure Time Activities Questionnaire [8].

Each criterion was assigned a score of 0 or 1. Participants were categorized into three different groups based on the total score: 0 as robust, 1 to 2 as prefrail, 3 or more as frail [8]. Sociodemographic and clinical data, including gender, age, marital status, living arrangements, education level, chronic conditions, and BMI was collected. The measures were taken by trained physiotherapists.

#### 2.3.2. Sleep Quality Measurements

The Pittsburgh Sleep Quality Index (PSQI) was used to examine sleep quality in the included sample. This scale consisted of 19 self-rated questions in 7 different domains, such as sleep quality, sleep duration, sleep latency, sleep disturbance, habitual sleep efficiency, daytime dysfunction, and sleep medications. The global PSQI score range from 0 to 21. A total score of 5 or more represents poor sleep quality; a higher score indicates poorer sleep quality.

### 2.4. Data Analysis

The STATA software 15.1 (Stata Corp, College Station, TX, USA) was utilized to analyze the data. The mean and standard deviation were calculated for continuous variables, and the frequency and percentage for categorical variables. Analysis of the variance for continuous variables and a chi-square test for categorical variables was used to compare the difference between baseline sociodemographic and clinical characteristics according to the frailty category. The relationship between sleep quality and the risk of frailty was investigated using various multiple logistic regression models. Adjusted and unadjusted odds ratios (OR), with the 95% CI were estimated. The level of statistical significance was defined at α < 0.01.

## 3. Results

A total of 270 participants were included in the main analysis. Table 1 shows the basic sociodemographic and clinical characteristics of the current sample according to the frailty category. The average age of our sample was 69 years old. About 65% of the current sample were male. Overall, a total of 29.2% of participants were classified as frail (females, 25.5%), 37.7% were pre-frail (females, 39.3%), and 32.9% were robust (females, 35.1%), as shown in Table 1.

The average PSQI score of the current study sample (aged 69.9 ± 6.2 years) was 5.9 ± 2.7. Overall, about 66% of the study sample had poor sleep quality with a PSQI score > 5. The pre-frailty and frailty status were prevalent among older adults who had poor sleep quality, 37% and 37.6% (*p* < 0.001), respectively (Table 1). The findings of multiple logistic regression analysis are presented in Table 2. Age and gender were adjusted for in the first model. The second model was adjusted for age, gender, BMI, Mini-Mental State Examination (MMSE) score, grip strength, living status, marital status, and gait speed. After adjusting for possible confounders, poor sleep quality, which was defined as a PSQI greater than 5, was maintained to be independently related to pre-frailty and frailty groups (OR = 1.67, 95% CI (1.26–2.05) (OR = 2.13, 95% CI (1.47–3.12), respectively.

## 4. Discussion

The aim of this study was to examine the relationship between sleep quality and the risk of frailty in the Saudi elderly population. Our results show that poor sleep quality (PSQI > 5) was independently related to frailty and prefrailty groups in our sample. The association was maintained as significant after adjusting for potential covariates. This is the first study in Saudi Arabia that looked at the association between sleep quality and frailty in an elderly population.

We found a prevalence of 65.9% of our sample had poor sleep quality. In our study, the prevalence of poor sleep quality is relatively high when compared to older adults in other nations, such as Thailand (44%), Japan (37%), China (49.7%), the United States (28%), and Korea (60%) [22,23,24,25,26]. The high prevalence in our study can be possibly attributed to the fact that poor sleep quality increased with age [27], and about 52% of our current sample was 70 years and older.

The present study found a link between poor sleep quality and frailty status among in both genders. After controlling for possible confounders, older individuals who scored higher than 5 on the PSQI were 1.67 and 2.13 times more likely to be classified as prefrail and frail, respectively. Previous studies from different countries reported similar findings. In the United States, data from the Osteoporotic Fractures in Men (MrOS) trial found that both subjective and objective sleep quality were independently associated with being frail (OR = 1.28) in both cross sectional and longitudinal analyses [28,29]. Shih et al., have reported a significant association between poor sleep quality, and prefrailty and frailty status in the Taiwanese elderly population (OR = 1.95) [30]. Our results were also consistent with a study conducted in China in which they found that older adults with poor sleep quality were twice as likely to be categorized as frail [31]. Moreover, our results were also similar to those in Ecuador, where researchers have found that higher scores in the PSQI were significantly associated frailty status in community-dwelling older adults [32].

However, the exact mechanism behind the association between sleep parameters and frailty is unclear. There are several probable underlying mechanisms that could explain the relationship between sleep quality and frailty. Empirical research shows that sleep disturbances may alter adipokines initially, increasing the risk of being frail through their direct impacts on adipokines. As a result, adipokines should be further investigated in the future as a potential mediator between sleep and the risk of frailty. Moreover, sleep disturbances may be indicators of comorbidities and poor health, which raises the risk of frailty [18]. Other studies found that inflammation and oxidative stress were possible contributors to frailty [33]. Furthermore, inflammatory processes might be a plausible mechanism through which sleep quality and frailty were related. Sleep-wake disruption influences inflammatory pathways, which impacts on the development of frailty. Inflammation is among the key pathophysiological alterations which might be linked to frailty [34]. One possible explanation was psychological disturbance, which explains how sleep quality affects frailty through insufficient physical activities. Prior research has indicated that poor sleep quality may lead to depression and a reduction in physical activities, raising the risk of developing adverse health outcomes [35,36,37]. A recent study showed that sleep disturbances may be associated with an increased incidence of sarcopenia. Sarcopenia causes a reduction in the muscle mass, decreased in coordination leading the individual to functional limitations and disability. Sarcopenia is important in the pathophysiology of frailty. Sleep problems appear to have an impact on sarcopenia and frailty via several pathways [38,39]. The underling pathophysiological mechanisms of association between sleep disturbances and frailty imply a bidirectional connection, and previous studies identify sleep disorders explicitly as a risk factor for frailty. Despite the fact that both diseases are modifiable, it has been suggested that studying sleep as a risk factor for frailty has greater practical clinical consequences.

This is the first research study to examine the correlation between sleep quality and frailty in a geriatric population in Saudi Arabia. There were several limitations in this study that should be considered when interpreting the findings. First, a causality relationship cannot be established between sleep quality and the risk of frailty due to the nature of the cross-sectional study design. Second, using a self-reported measure of sleep may have resulted in recall bias. Even though it has an excellent psychometric property, objective measures, such as actigraphy, offer more in-depth knowledge of sleep quality. Third, the small sample size plus the current sample was recruited from a single geographical region in Saudi Arabia, therefore the generalizability of our findings may be limited.

Given the association between sleep quality and frailty, and the negative effect of poor sleep quality on older adults’ health, a regular sleep assessment should be considered. In addition, healthcare providers should consider implementing a sleep educational program for older adults. Other non-medical interventions, such as exercises, should be recommended to improve sleep quality in older adults.

## 5. Conclusions

Our data showed a high prevalence of poor sleep quality among Saudi older individuals. Poor sleep quality measured by PSQI was independently related to both pre-frailty and frailty. Because the associations were based on a cross-sectional study, the influence of sleep disturbances on the risk of frailty should be verified in longitudinal studies with more generalizable samples.

## Figures and Tables

**Table 1:** Sociodemographic and clinical characteristics of the study sample according to frailty status.

Variable	Total Sample N = 270 (%)	Frailty Status, n (%)			*p*
		Robust	Pre-Frail	Frail	
**Age groups ^a^**					
**60–69**	129 (47.6)	36 (27.9)	71 (55)	22 (17)	<0.001
**≥70**	141 (52)	53(37.5)	31 (22.6)	57 (39.7)	
**Gender ^a^**					
**Male**	176 (65.2)	56 (31.8)	65 (36.9)	55 (31.2)	0.503
**Female**	94 (34.8)	33 (35.1)	37 (40.4)	24 (24.4)	
**Education ^a^**					
**Primary school**	160 (59)	55 (34.4)	57 (35.6)	48 (30)	<0.001
**Secondary** **school**	68 (25.1)	35 (51.4)	23 (35.2)	10 (13.2)	
**College**	42 (15.5)	21 50)	13 (30.9)	8(19)	
**Marital status ^a^**					
**Married**	169 (62.4)	64 (37.9)	67 (39.6)	38 (22.4)	<0.001
**Single/widowed/divorced**	101 (37.3)	25 (24.7)	35 (35.6)	41 (39.6)	
**Living arrangement ^a^**					
**Living with others**	237 (87.5)	82(34.5)	89 (37.5)	66 (27.8)	0.051
**Living alone**	33 (12.2)	7(21.2)	13 (42.4)	13 (36.S)	
**Number of chronic conditions ^a^**					
**<3**	183 (67.7)	64 (34.9)	81 (44.2)	38 (20.7)	<0.001
**≥** **3**	87(32.2)	25 (28.7)	21 (25.2)	41 (45.9)	
**Grip strength, mean (SD)**	270	24.66 (12.2)	21.01 (11.02)	20.2 (7.5)	<0.001
**BMI (kg** **m^−2^), mean (SD) ^b^**	236	27.8 (4.4)	26.5 (4.8)	24.4 (5.5)	<0.001
**MMSE score ^a^**					
**Normal (>24)**	182 (67.4)	74 (40.6)	76 (2.3)	32 (17.1)	<0.001
**Impaired (<24)**	88 (32.5)	15 (17.1)	26 (29.5)	47 (53.4)	
**Subjective sleep quality** **^a^**					
**PSQI score ≤ 5**	92 (34.1)	44(47.8)	36 (39.1)	12 (13.1)	<0.001
**PSQI score > 5**	178 (65.9)	45 (25.2)	66 (37.1)	67 (37.6)	
**Mean PSQI score (SD)**		5.8 (2.3)	7.3 (1.6)	8.1(2)	

BMI: body mass index, MMSE: Mini-Mental State Examination, SD: standard deviation, PSQI: Pittsburgh sleep quality index; ^a^: Chi-square test, ^b^: One-way ANOVA.

**Table 2:** Association between sleep quality and frailty status.

Subjective Sleep Quality	Crude Model		Model 1		Model 2	
	OR	95% CI	OR	95% CI	OR	95% CI
**Pre-frail**	Ref.		Ref.		Ref.	
**PSQI score ≤ 5 (ref)**						
**PSQI score > 5**	1.83 *	(1.1–3.2)	1.73 *	(1.42–2.12)	1.67 *	(1.26–2.05)
**Frail**	Ref.		Ref.		Ref.	
**PSQI score ≤ 5 (ref)**						
**PSQI score > 5**	2.44 *	(1.28–4.63)	2.21 *	(1.18–4.11)	2.13 *	(1.47–3.12)

Crude model: crude multiple regression model. Model 1: Multiple regression after adjusting for age and gender. Model 2: Multiple regression after adjusting for age, gender, BMI, MMSE score, grip strength, living status, marital status, and gait speed. OR: odds ratio; 95% CI: confidence interval; * *p* < 0.01.

## Data Availability

The datasets used and/or analyzed during in the current study are available from the corresponding author on reasonable request.
